# Supplementary material for: Patient and clinician perspectives of a remote monitoring program for COVID-19 and lessons for future programs
Source: BMC Health Serv Res. 2023 Jun 27;23:698. doi: 10.1186/s12913-023-09684-1 (PMC10304230; doi:10.1186/s12913-023-09684-1)
Supplement: Supplementary file 1 — Additional file 1. [file 12913_2023_9684_MOESM1_ESM.docx]

**S1. Interview Guide for Patients**

**PATIENT’S COVID EXPERIENCE**

Thank you for taking the time to participate in this interview about your experience with COVID Watch. As mentioned in our Informed Consent Overview, our goals for this interview are to understand your experience in the program, and in particular to understand how the program may have affected your ability to manage COVID at home as well as your decisions about your health care. We’re also interested in understanding why you chose to participate in the program. Finally, we’d like to hear any thoughts you have about improving the COVID Watch experience.

Some questions may bring up difficult experiences for you; if at any time you feel that you would like a break, just let me know.

We’ve already completed your informed consent verbally. I’d also like to record the interview, to make sure we don’t miss anything that you say. Are you OK with this interview being recorded?

Great! I’ll turn the recorder on now. *[State Interviewer Initials, Patient ID, Project Name, Date.]*

We’ll start with some questions about your experience of COVID.

Think back to when you first learned that you had COVID.

1. What was your health like in general at the time you first noticed symptoms What kinds of symptoms did you experience that made you feel the need to get a COVID-10 test?
   - What about your health made you concerned enough that you got tested?
   - What kind of symptoms did you experience that you feel the need to get a COVID-19 test?
   - Do you have any health conditions that affected your experience of COVID? For example, we know some people have certain conditions (like asthma) that put them at higher risk of certain COVID symptoms.
2. What it was like when you first found out you had COVID?
   - How were you feeling emotionally?
     - *Probe for feelings of worry, stress, or lack thereof if not mentioned*
   - Getting a positive COVID-19 diagnosis can be stressful; what was your level of stress when you found out you were positive?
     - *If not worried or stressed*: What made you feel less stressed/worried about the situation?
3. How are you feeling now?

Some people say they had trouble accessing care or being able to seek medical care.

1. What was your experience with accessing medical care?

I want to get a sense of your experience after the first few days...

1. What was the worst time point of your COVID symptoms?
   - What about that made it the worst point for you?
   - About how many days after you were diagnosed did these symptoms peak?
2. How would you have rated your COVID symptoms on a scale between 1 and 10? 1 being pretty minimal to 10 being the worse you could have ever imagined.

**PATIENT’S COVID WATCH PERCEPTIONS**

Now let’s talk about COVID Watch, the program that texted you in the morning and afternoon to ask you how you were doing.

1. What was your experience using COVID Watch like?
   - What did you think of the program overall?
2. What were your main reasons for staying enrolled in the program? (Or responding to messages?)
3. What did you think of the text messages you received?
   - Did the texts make sense to you?
   - *If need clarification*: Were you able to easily understand them? Were you able to understand how you were supposed to respond?
   - *If no,* What about the texts was confusing/didn’t make sense?
   - *[Spanish-speakers only]*: can you tell me about the messages you received in Spanish? Was the translation (or language) easy to understand?
4. Did a nurse ever call you regarding your text answers? (*If “no” move on to question 11*)
   - If YES:
     - What do you think made it so they felt they should contact you?
     - What was that phone call like? What did they instruct you to do?
     - What did you think about the experience of being referred to a nurse?
   - If NO
     - If you can remember, were you ever expecting a call from a nurse but didn’t receive it?
       - If YES, tell me more about the situation. What happened?
       - If NO, move to next questions.

If they were a LOW user (*high users skip*)

1. We noticed that you stopped responding to texts or left the program before 10 days. That was common for other people in the program. Can you tell me what made you stop responding to COVID watch texts?
2. How did you feel when you got the text messages each time from the program.
   - What made you feel that way?

If they were a HIGH user (*low users skip*)

1. Tell me how you felt when you got the text messages each time from the program.
   - What made you feel that way?
2. When you didn’t respond to the texts, what were some of the reasons?
   - What did you think about the frequency of the messages?
     - *If not addressed, probe:* Were they too frequent, just frequent enough, or not frequent enough?) What makes you think that?

If they received a PULSE OX (*if no pulse ox, skip*)

You received a pulse oximeter device from COVID Watch, the device that goes on your finger and tells you your oxygen level.

1. On average, how often did you use the pulse ox? Why?
2. How did knowing your blood oxygen level make you feel about your COVID experience?

Wrap it up

1. Now I’d like to take a step back, to think more generally about your experience with COVID Watch. What role did COVID Watch play in helping you manage your symptoms, if any?
2. How do you think it changed the way you experienced COVID, if it did?
3. What kinds of changes do you think the program needs to make to be more useful for patients in the future?

**SUPPLEMENTARY INFORMATION**

I’m going to ask you a few other questions to try and better understand the care you received while you had COVID, including anything beyond just the scope of COVID Watch.

1. Can you tell me about other interactions or visits you had with nurses or doctors during your COVID illness?
   - Did you see a doctor in person? Tell me...where did you go? How many times did you go?
   - Did you have any phone conversations with a nurse or doctor not through the COVID Watch program?
     - Tell me more: Who did you call? What about? Any more? *(repeat who and what about)*
   - Did you have any telemedicine appointments?
     - Tell me more: With whom? What about? Any more? *(repeat who and what about)*

Some people have devices at home that help them collect information about their health like their oxygen level, heart rate, or blood pressure.

1. Do you have one or any of those devices at home? (*If yes, continue*)
   - What kind?
   - Did you use it while you had COVID?
   - Did you find those devices helpful? Why do you say that?

Is there anything that we didn’t discuss today that you think we should know about your experience with COVID Watch?
