# Supplementary material for: Patient and clinician perspectives of a remote monitoring program for COVID-19 and lessons for future programs
Source: BMC Health Serv Res. 2023 Jun 27;23:698. doi: 10.1186/s12913-023-09684-1 (PMC10304230; doi:10.1186/s12913-023-09684-1)
Supplement: Supplementary file 2 — Additional file 2. [file 12913_2023_9684_MOESM2_ESM.docx]

## **S2. Interview Guide for Clinicians**

## **Interview Guide (Primary Care Clinicians)**

Thank you for taking the time to participate in this interview. Our goal for this interview is to understand your experience with the COVID Watch program as a Penn Medicine Clinician. COVID Watch is a Penn Medicine monitoring program that automatically checks in with patients self-isolating at home, diagnosed with COVID-19 or had symptoms that could be concerning for COVID-19.

Participation in this study is completely voluntary and you can withdraw at any time. Please know that everything you say today is confidential. The things you share will not be connected to your name, and the recording will be destroyed after it is transcribed. Any identifying information, for example your name or your patients’ names, will be removed from the transcript. We request that you allow us to audio-record the conversation as it will ensure we capture your thoughts and views completely.

Ask: Do you have any questions about the study or what is required to participate? (Answer all questions).

Ask: Are you comfortable with me recording this conversation? (Pause for confirmation).

1. To start, please tell me about your clinical role at Penn...
   1. What is your job title or role?
   2. Where do you interact with patients?
   3. How many clinical hours do you typically work in a week?
      1. *Probe if necessary:* In your estimate, how many [days or hours] that would be?
   4. *Ask:* What percentage of your time is clinical?

1. As we mentioned, we are interested in learning more about the COVID Watch program. A number of Penn Medicine patients were enrolled in the COVID Watch program. Some sites and providers were high enrollers, while others were low enrollers. Tell us, what led you or your clinical team to use or not use COVID Watch?
   1. Think back to patients with COVID, what factors did you consider when you thought about enrolling in COVID Watch or not?
   2. What were the characteristics of patients or things about the patient that influenced your decision to enroll them in COVID Watch or not? Such as: Things in their home life? Things about their medical conditions or social needs? What about their ability to access medical care?
   3. What were the factors related to your work environment or your department—for example, your department’s culture or your colleagues-- that influenced your to enroll them in COVID Watch or not?
   4. In addition to these reasons, did you have any other personal reasons that factored into your decision to enroll patients in COVID Watch?
   5. [If not already discussed] Tell me about the process of actually enrolling a patient in COVID watch. What was that like? *(Probe: what, if anything, made it easy or hard?)*
2. Now I want you to think about two patients - one you enrolled in COVID Watch and another you didn’t enroll...
   1. For the patient you enrolled, what led you to enroll the patient in COVID Watch?
   2. For the patient you did NOT enroll, what led you to not enroll the patient in COVID Watch?
3. What was it like to have a patient in COVID Watch?
   1. Prompt: How did you manage COVID Watch enrolled patients differently than individuals not in COVID Watch, if at all?
   2. Prompt: How did you know they were in COVID Watch?
   3. From what you recall, did you have any patients who escalated to the COVID Watch nurses?
      1. If YES
         1. Based on those patients, what level of confidence or lack thereof did you have with the clinical judgement of nurses and providers supporting the COVID Watch program?
         2. Can you tell me about a time when you felt you would have managed their care differently than the COVID Watch team? If YES - Tell me more?
      2. If NO: move on to the next question

1. What recommendations do you have for improving COVID Watch?
   1. Prompt: What would have made it more useful to you?
   2. Prompt: Tell me about any frustrations or difficulties with any aspect of it.
   3. Prompt: What other thoughts do you have about the process, for example about things like the amount of time it took, or the ease of enrolling?
2. More and more health systems are considering remote patient monitoring programs, like COVID Watch, for both acute and chronic conditions. Can you share any “lessons learned” you have had from your experience with COVID Watch that might be relevant for future remote patient monitoring programs?
   1. How do you think remote patient monitoring programs could influence your clinical practice in the future? Are there particular areas or conditions that you think remote patient monitoring is most useful for?
   2. What parts of COVID Watch’s remote patient monitoring program do you think was the most useful for patients? For you?
   3. What aspects of COVID Watch do you think will be important for future remote patient monitoring programs?
3. The COVID-19 pandemic has highlighted some of the racial and socioeconomic disparities present in our country in many different ways. In particular, this is true in terms of COVID testing and treatment and the overall impact of COVID.
   1. What effects do you think COVID Watch might have had on these racial and class disparities?
      1. *Probe*: How might have COVID Watch worsened the disparities? How might it have lessened them?
   2. Overall, apart from racial and class disparities, are there patients who you feel benefited from or were disadvantaged by COVID Watch more than others?
4. We are interested in talking to other people who may have been involved in enrolling people into COVID Watch. Who are the other members of your team (e.g., NP, nurses, or case managers) we should be talking to?

Is there anything that we didn’t discuss today that you think we should know about your experience with COVID Watch?

Thank you for taking the time to speak with us today.

##

## **Interview Guide (ED Clinicians)**

Thank you for taking the time to participate in this interview. Our goal for this interview is to understand your experience with the COVID Watch program as a Penn Medicine Clinician. COVID Watch is a Penn Medicine monitoring program that automatically checks in with patients self-isolating at home, diagnosed with COVID-19 or had symptoms that could be concerning for COVID-19.

Participation in this study is completely voluntary and you can withdraw at any time. Please know that everything you say today is confidential. The things you share will not be connected to your name, and the recording will be destroyed after it is transcribed. Any identifying information, for example your name or your patients’ names, will be removed from the transcript. We request that you allow us to audio-record the conversation as it will ensure we capture your thoughts and views completely.

Ask: Do you have any questions about the study or what is required to participate? (Answer all questions).

Ask: Are you comfortable with me recording this conversation? (Pause for confirmation).

1. To start, please tell me about your clinical role at Penn...
   1. What is your job title or role?
   2. Where do you interact with patients?
   3. How many clinical hours do you typically work in a week?
      1. *Probe if necessary:* In your estimate, how many [days or hours] that would be?
   4. What percentage of your time is clinical?

1. As we mentioned, we are interested in learning more about the COVID Watch program. A number of Penn Medicine patients were enrolled in the COVID Watch program. Some sites and providers were high enrollers, while others were low enrollers. Tell us, what led [you or your clinical team] to use or not use COVID Watch?
   1. What were the **characteristics** of patients or things about the patient that influenced whether [you or your team] enrolled a patient in COVID Watch?
      1. [*Probe if needed]* Such as: Things in their home life? Things about their medical conditions or social needs? What about their ability to access medical care?
   2. What were the factors related to your **work environment** – like your colleagues, or your practice’s culture -- that influenced your decision about whether or not to enroll a patient in COVID Watch?
      1. How are patients who receive a positive COVID test **after** discharge from the ED managed in your Emergency Department?
      2. How has that changed over the course of the pandemic?
   3. In addition to these reasons, did you have any other **personal reasons** that affected whether you enrolledpatients in COVID Watch or not?
      1. How does the presence (or absence) of **automated test results** reporting and automated enrollment in COVID Watch influence your clinical decision making?
   4. [If not already discussed] Tell me about the process of actually enrolling a patient in COVID watch. What was that like?
      1. *(Probe: What, if anything, made it easy or hard?)*

Next, we know a lot of patients are sent home and a few get admitted. We are really interested in those patients right on the cusp-- where COVID Watch would have been a viable option, and what pushed you one way or the other.

1. Think about a patient you felt comfortable discharging home on COVID Watch. What did COVID Watch provide that made you feel comfortable sending them home?
2. *[Probe if needed:]* What made you feel uncomfortable about sending them home without it? For patients whom you discharged and enrolled in COVID Watch-- do you think in you would have discharged any of these patients **even if** COVID Watch had been available?
   1. *[Probe if necessary]* Why did you choose to enroll them in COVID Watch?
   2. *[Probe if necessary]* How often do you think this happened?
3. There was also a program in the ED called COVID Pulse, in which patients were discharged with a home pulse oximeter. COVID Watch did not provide patients with a home pulse oximeter.
   1. Did you enroll patients in the COVID Pulse program specifically?
      1. IF YES: If so, how did you decide to enroll a patient in COVID Watch vs. COVID Pulse?
      2. Did you feel any more or less comfortable discharging patients on COVID Watch vs. COVID Pulse? If so, tell me more.
4. Moving from discharging patients to when patients arrived in the ED: Do you recall seeing any patients who were already in COVID Watch when they arrived in the ED, or had been sent to the ED by COVID Watch?
   1. If YES: How did their being sent by COVID Watch impact your assessment or treatment of them, if at allt?
      1. *Probe:* How did the information provided or documented by the COVID Watch team impact your treatment plan, if at all?
   2. If YES: Do you recall patients having or reporting the use of a home pulse oximeter device, like the one used on a finger?
      1. If YES: Can you tell me more about how the pulse oximeter might have impacted your assessment and treatment of the patient?
      2. If NO: Move on to the next question
   3. If NO: Move on to the next question
5. Zooming out, with your experiences in mind: what **recommendations** do you have for improving COVID Watch?
   1. *Probe*: What, if anything, about COVID Watch could have been changed to have helped you avoid hospitalizing patients? (E.g. phone calls, increased monitoring, etc.)
   2. *Probe*: What would have made it more useful to you?
   3. *Probe*: Tell me about any frustrations or difficulties with any aspect of it.
   4. *Probe*: What other thoughts do you have about the process, for example about things like the amount of time it took, or the ease of enrolling?
6. Now, thinking about the future more broadly, more and more health systems are considering remote patient monitoring programs, like COVID Watch, for both acute and chronic conditions. Can you share any “**lessons learned**” you have had from your experience with COVID Watch that might be relevant for future remote patient monitoring programs?
   1. How do you think remote patient monitoring programs could influence your clinical practice in the **future**?
      1. Are there particular areas or conditions that you think remote patient monitoring is most useful for?
   2. What parts of specifically COVID Watch’s remote patient monitoring program do you think was the **most useful** for patients? For you?
   3. What aspects of COVID Watch do you think will be important for future remote patient monitoring programs?
7. Finally, we know that the COVID-19 pandemic has highlighted some of the racial and socioeconomic disparities present in our country in many different ways. In particular, this is true in terms of COVID testing and treatment and the overall impact of COVID.
   1. What effects do you think COVID Watch might have had on these racial and class disparities?
      1. *Probe*: How might COVID Watch have worsened the disparities? How might it have lessened them?
   2. Overall, apart from racial and class disparities, are there patients who you feel benefited from or were disadvantaged by COVID Watch more than others?

We are interested in talking to other people who may have been involved in enrolling people into COVID Watch. Who are the other members of your team (e.g., NP, nurses, or case managers) we should be talking to?

1. Great! Thank you so much for sharing your thoughts. We’ve covered all of the areas I wanted to cover with you. Is there anything else about your experience with COVID Watch that you think we should know?
