# Supplementary material for: Patient and clinician perspectives of a remote monitoring program for COVID-19 and lessons for future programs
Source: BMC Health Serv Res. 2023 Jun 27;23:698. doi: 10.1186/s12913-023-09684-1 (PMC10304230; doi:10.1186/s12913-023-09684-1)
Supplement: Supplementary file 3 — Additional file 3. [file 12913_2023_9684_MOESM3_ESM.docx]

## **S3. Interview Guide for Administrators**

Thank you for taking the time to participate in this interview. Our goal for this interview is to understand your experience with the COVID Watch program as a key leader, manager, or administrator within Penn Medicine. COVID Watch is a Penn Medicine monitoring program that automatically checks in with patients self-isolating at home, diagnosed with COVID-19 or had symptoms that could be concerning for COVID-19.

Participation in this study is completely voluntary and you can withdraw at any time. Please know that everything you say today is confidential. The things you share will not be connected to your name, and the recording will be destroyed after it is transcribed. Any identifying information, for example your name or your patients’ names, will be removed from the transcript. We request that you allow us to audio-record the conversation as it will ensure we capture your thoughts and views completely.

Ask: Do you have any questions about the study or what is required to participate? (Answer all questions).

ASK: Are you comfortable with my recording this conversation? [*Turn recorder on*]

1. To start, please tell me about your role at Penn...
   1. What is your job title or role?
   2. Do you interact with patients in any capacity?
      1. If YES: In what way?
      2. If YES, and clinical: Roughly what percentage of your time is clinical?

Next, we're going to be asking about your practice or department’s experience with managing COVID-19 patients within two distinct time periods: (a) towards the beginning of the pandemic and (b) more recently.

1. ED or PCP:
   1. **ED**: I’d like for you to think back to the first few months of the pandemic, when a patient’s COVID test came back positive, or when there was a clinical suspicion for COVID. What factors went into how your department decided whether to admit or discharge these patients?
   2. **PCP**: In the first couple of months of the pandemic, when a patient’s COVID test came back positive, or when there was a clinical suspicion for COVID, what factors did your practice consider when deciding how these patients would initially receive care?
   3. *Prompt (if Primary Care related practice/department)*: How did your practice or department decide when they would need to be seen in-person, such as a clinic, urgent care center, or go to the emergency department?
   4. **Both:** Do you think Covid Watch affected your practice’s or department’s decision-making about how patients received care? In what ways, if at all?
2. Now I’d like for you to think about your [department/practice] current care patterns.
   1. How has your [practice or department’s] approach to managing patients with a positive COVID test (or patients whom your clinician’s strongly suspected having COVID) changed, if at all?
      1. **For ED:** How are patients who receive a positive COVID test after discharge from the ED managed in your Emergency Department, if at all?
      2. How has this changed over time (if at all)? How was it in the beginning of the pandemic compared to today?
   2. Does Covid Watch**currently** impact your current decision-making about care? In what ways, if at all?

Now I want you to think more generally about how your [team or practice] manages the needs of patients with COVID or patients you thought might have COVID....

1. A number of Penn Medicine patients were enrolled in the COVID Watch program. Some practices and some sites referred a lot of patients and some didn't. We’d like you to help us interpret this finding...

Tell us, what influenced your [practice or department] to enroll (or not enroll) patients in COVID Watch?

(Probe if need be: In general, what do you think might have kept providers from using COVID Watch? Conversely, what might have made COVID Watch appealing to use?)

- - 1. More specifically, what were the **characteristics** of patients or things about the patient that influenced whether or not [you or your team] enrolled a patient in COVID Watch?
    2. [*Probe if needed]* Such as: Things in their home life? Things about their medical conditions or social needs? What about their ability to access medical care?
    3. What were the factors related to your **work environment** —like your practice’s culture, or your colleagues-- that influenced whether you used or enrolled patients in COVID Watch (e.g. your colleagues, or your practice’s culture)? What sorts of communication occurred to encourage enrollment in COVID Watch? Who were they from? How frequent were they? Were they effective? Did you specifically communicate or encourage the use of COVID Watch? Tell me more.
    4. In addition to these reasons, were there any **personal reasons** that might have influenced your [practice’s or department’s] decisions to enroll patients in COVID Watch or not?
    5. [If not already discussed] What are your thoughts on the **process** of actually enrolling a patient in COVID watch. *Probe: what, if anything, made it easy or hard?)*
    6. For ED: How does the presence (or absence) of automated results reporting and automated enrollment in COVID Watch influence this process?

1. With your experience in mind, what **recommendations** do you have for improving COVID Watch?
   1. For ED: What, if anything, could have been different about it that would have prevented needing to hospitalize patients?
   2. Prompt if need be: What would have made it more useful to your practice or department?
   3. Prompt if need be: Tell me about any frustrations or difficulties with any aspect of it.
   4. Prompt if not discussed yet: What other thoughts do you have about the process, for example about things like the amount of time it took, or the ease of enrolling? What about continuity of care or care fragmentation?
2. Looking to the future, we know that more and more health systems are considering remote patient monitoring programs, like COVID Watch, for both acute and chronic conditions. Can you share any “**lessons learned**” or insights you or your practice have had using COVID Watch that might be relevant for future remote patient monitoring programs?
   1. How do you think remote patient monitoring programs could influence your [practice or department] in the **future**? Are there particular areas or conditions that you think remote patient monitoring is most useful for?
   2. What parts of specifically COVID Watch’s remote patient monitoring program do you think were the **most usefu**l for patients? For you or your practice?
   3. What aspects of COVID Watch do you think will be **important** for future remote patient monitoring programs?
3. Zooming out, we also know that the COVID-19 pandemic has highlighted some of the racial and socioeconomic disparities present in our country, in many things but particularly related to COVID-19 testing, treatment, and the overall impact of COVID.
   1. What effects do you think COVID Watch might have had on these racial and class disparities?
      1. *Probe*: How might COVID Watch have worsened the disparities? How might it have lessened them?
   2. Overall, apart from racial and class disparities, are there patients who you feel benefited from or were disadvantaged by COVID Watch more than others?
4. We are interested in talking to other people who may have been involved in enrolling people into COVID Watch. Who are the other members of your team (e.g., nurses, or case managers) we should be talking to?

Great! Thank you so much for sharing your thoughts. We’ve covered all of the areas I wanted to cover with you. Is there anything else about your experience with COVID Watch that you think we should know?
